# Natural-Synthetic Hybrid Polymers Developed via Electrospinning: The Effect of PET in Chitosan/Starch System

**DOI:** 10.3390/ijms12031908

**Published:** 2011-03-16

**Authors:** Adolfo Espíndola-González, Ana Laura Martínez-Hernández, Francisco Fernández-Escobar, Victor Manuel Castaño, Witold Brostow, Tea Datashvili, Carlos Velasco-Santos

**Affiliations:** 1 College of Engineering, National Autonomous University of Mexico, Edificio Bernardo Quintana, Cd. Universitaria, CP. 04510, Mexico D.F., Mexico; E-Mail: ameg00@hotmail.com; 2 Centre of Applied Physics and Advanced Technology, National Autonomous University of Mexico, Campus Juriquilla, Querétaro, Mexico; E-Mails: francisco@fata.unam.mx (F.F.-E.); castano@fata.unam.mx (V.M.C.); 3 Queretaro Institute of Technology, Av. Tecnológico S/N Esq. Gral. Mariano Escobedo, Col. Centro Histórico. CP. 76000, Queretaro, Mexico; E-Mail: analaura@fata.unam.mx; 4 Laboratory of Advanced Polymers & Optimized Materials (LAPOM), Department of Materials Science & Engineering and Center for Advanced Research and Technology, University of North Texas, UNT Discovery Park Room E-132, 3940 N. Elm. Denton, TX 76207, USA; E-Mails: brostow@unt.edu (W.B.); tea_datashvili@yahoo.com (T.D.)

**Keywords:** natural-synthetic, chitosan, molecular interactions, electrospinning, PET

## Abstract

Chitosan is an amino polysaccharide found in nature, which is biodegradable, nontoxic and biocompatible. It has versatile features and can be used in a variety of applications including films, packaging, and also in medical surgery. Recently a possibility to diversify chitosan properties has emerged by combining it with synthetic materials to produce novel natural-synthetic hybrid polymers. We have studied structural and thermophysical properties of chitosan + starch + poly(ethylene terephthalate) (Ch + S + PET) fibers developed via electrospinning. Properties of these hybrids polymers are compared with extant chitosan containing hybrids synthesized by electrospinning. Molecular interactions and orientation in the fibers are analyzed by infrared and Raman spectroscopies respectively, morphology by scanning electron microscopy and thermophysical properties by thermogravimetric analysis and differential scanning calorimetry. Addition of PET to Ch + S systems results in improved thermal stability at elevated temperatures.

## 1. Introduction

Attempts to create new polymeric materials are based more and more on renewable sources, with the objective to diminish the use of synthetic petroleum-based raw materials. Natural polymers have diverse advantages; they are environmentally friendly, nontoxic, biodegradable and some even exhibit antibacterial activity. In spite of these characteristics, poor mechanical and thermal properties of biopolymers limit their applications. One way to overcome the disadvantages is via combination of synthetic and natural polymers. We thus acquire biofunctionalities of biopolymers together with easy modification and processability of synthetic polymers.

This type of novel polymer could be called natural-synthetic hybrid materials and are based on the synergic effect between synthetic and biopolymeric constituents. Naturally occurring polymers include: proteins, nucleic acids and polysaccharides. These last are strongly bioactive and are generally derived from agricultural, feedstock or crustacean and shell wastes [1], therefore constitute an abundant and renewable source. Particularly chitosan, a biocompatible and biodegradable polysaccharide mainly used in biomedical and cosmetics applications, has also potential as an edible packaging polymer or coating; it has an ability to form films without any use of additives [2]. Chitosan has been used with other types of polysaccharides such as the starch to render specific properties like water vapor barrier characteristics [3]. Combination of chitosan with synthetic polymers is not easy, since high melting temperatures are required in the processing of these latter while temperatures over 200 °C cause chitosan degradation. Diverse methods such as grafting [4], chemical functionalization [5], wet spinning [6], chemical cross linking [7], casting [8], melt blending [9] and electrospinning [10] have been used to produce hybrid materials based on chitosan and synthetic polymers. In particular, electrospinning is a relatively new technique that has been used to manufacture nano and micro fibers [11].

We have used electrospinning to synthesize hybrid materials based on chitosan, starch and poly(ethylene terephthalate) (PET). Chitosan can be advantageously used in electrospinning due to its physicochemical properties; it is soluble in organic acids such as dilute aqueous acetic acid, formic and lactic acids, also in mixtures of water with methanol, ethanol or acetone. This biopolymer has free amino groups which make it a positively-charged polyelectrolyte in acidic pH [10]. Chitosan’s positive charges allow it to have strong electrostatic interactions with negatively charged molecules [12]. In addition, hydrogen bonds in chitosan solutions facilitate the formation of microfibrils.

On the other hand, PET is widely used due to its transparency, good impact resistance, low density and easy processability [13]. Thus, combining PET and chitosan could result in a new kind of hybrid materials, where properties of both polymers act synergically, providing a novel material more biodegradable and less harmful to the environment. However, combination of PET and chitosan is not so easy, since both processing temperature and policondensation reaction temperature (280 °C) [14] are inappropriate for working with chitosan. This means that neither melt-blending nor straightforward *in situ* polymerization can be employed to process PET together with chitosan to form hybrid polymers. Therefore, in the present project the solubility characteristics of both polymers are used to synthesize novel hybrid fibers where high temperatures are not required. Several hybrid systems based on chitosan have been synthesized by electrospinning with interesting structural and thermal properties. Some of them involve chitosan only [10,15]; poly(ethylene oxide) [12]; polycaprolactone [16]; poly(vinyl alcohol) [17]; polyacrylamide [18]; nylon 6 [19]; and PET [13]. Given these results, we have decided to use starch because of its chemical structure unlike other polymers used before and because of its biodegradability. We have studied molecular interactions, conformations, morphological and thermal behavior of chitosan + starch + PET systems (Ch + S + PET).

## 2. Experimental

### 2.1. Electrospinning Process

**Figure 1.**
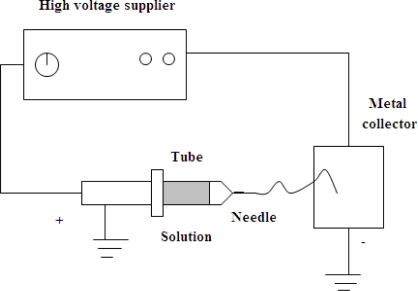
A schematic of electrospinning.

The electrospinning equipment includes three main components: a high voltage supplier, a capillary tube with a pipette or a needle of small diameter, and a metal collecting screen. One electrode is placed into the polymer solution and the other is attached to the collector (Figure 1). The process is initiated when a high voltage is applied; this causes the electrostatic force in the polymer solution to overcome the surface tension and creates an electrically charged jet of polymer solution out of the pipette. The solution jet passes a controlled distance between the pipette tip and the collector. Before reaching the collecting screen the solution jet evaporates, leaving behind charged polymer fibers that are assembled on the screen as an interconnected web of small fibers [10,11].

The electrospinning process has certain critical parameters, in particular an appropriate selection of the solvent system. In fact, solvent performs two crucial roles, one is to solvate the polymer molecules ready to form the electrified jet and the other is to carry the solvated polymer molecules toward the collector [15]. Other important parameters are voltage level, internal diameter of the tip and the tip-collector distance.

### 2.2. Electrospinning of Hybrid Fibers

In order to synthesize hybrid fibers of chitosan (Ch), starch (S) and PET by the electrospinning process, one begins by preparation of appropriate polymer and biopolymer solutions. First, chitosan (85% deacetalization degree), 7 wt%, is dissolved in an aq. acetic acid solution (90%). Chitosan is so dispersed under stirring until a gel is formed. On the other hand, aq. corn starch solution (also 7 wt%) is prepared by adding sorbitol as a plasticizer (1 wt%). After mixing the three components: water, corn starch and sorbitol, one heats the mixture for 10 minutes up to 90 °C until onset of gel formation. Then the solution is cooled to room temperature. A PET containing solution is then prepared using *o*-chlorophenol as the solvent. Commercial PET pellets and *o*-chlorophenol are introduced into a reflux system, temperature is maintained between 80 °C and 120 °C. The final concentration of PET in the solution is 3 wt%. All reagents used were received from Sigma Aldrich.

Solutions were prepared so as to determine the effects of varying the PET concentration on properties of the resulting hybrid fibers. Thus, several blends were developed in order to select concentrations that provide the best properties of the fibers. The selected percentages of chitosan and starch were: 85 and 15 wt% and also 70 and 30 wt%. These two blends were mixed with PET solutions so as to obtain 5 and 15 wt% PET. The final blends containing chitosan, starch and PET were placed into a commercial syringe with a stainless steel needle of 2 mm diameter. Table 1 shows the corresponding concentrations and nomenclature used in the synthesis and characterization of our ternary hybrid fibers. Moreover, a pure chitosan solution was electrospun so as to compare fibers so obtained with our ternary ones.

The synthesis of hybrid fibers was carried out by electrospinning technique using a high power supplier of 20 kV DC, 30 mA and 600 W. The tip-collector distance was 15 cm. A grounded copper collecting screen was used.

### 2.3. Characterization Techniques

Infrared spectroscopy was performed using a FTIR Brukker Vector 33 spectrophotometer with Attenuated Total Reflectance (ATR) mode, in the region from 400 to 4000 cm^−1^, spectral resolution of 1 cm^−1^ and 32 scans. Raman spectroscopy was performed in a Raman Senterra Bruker machine with a 100× microscope objective, laser emitting at 785 nm and resolution 0.1 cm^−1^. The morphology of the hybrid fibers was observed by SEM using a JSM-6060LV JEOL microscope at an accelerating voltage of 10 kV. For SEM study the samples were mounted on metal stubs and were vacuum-coated with gold at 7 × 10^−2^ mbar using argon in a sputter coater EMS 550. Thermogravimetric analysis (TGA) and differential scanning calorimetry (DSC) were carried out in a TA Instruments Q600 SDT using a scan speed of 10 °C/min and nitrogen as the purge gas.

### 3. Results and Discussion

### 3.1. Fourier Transform Infrared Spectroscopy

**Figure 2.**
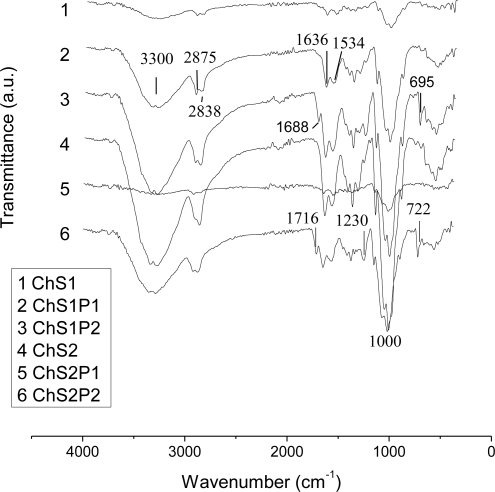
FT-IR spectra of hybrid fibers.

FT-IR spectroscopy was used to examine the interaction between chitosan, starch and PET in the hybrid fibers; the spectrograms are displayed in Figure 2. Typical bands for the natural polymers, chitosan and starch, are found at 3300 cm^−1^ (O–H stretching), 2875 cm^−1^, 2838 cm^−1^ (C–H symmetric/asymmetric stretching) and 1000 cm^−1^ (C–O deformation). Characteristic groups of chitosan appear in 1636 cm^−1^ (C=O amide I stretching) and 1534 cm^−1^ (N–H amide II stretching). The IR bands of the synthetic polymer (PET) are seen at: 1688 cm^−1^, 1230 cm^−1^ (C=O ester groups) and 695 cm^−1^ (benzene rings). Absorption bands of natural and synthetic polymers are observed due to the integration of the three components (chitosan, starch and PET) in the hybrid. Common interactions in hybrid polymers based on chitosan occur between the primary hydroxyl groups of chitosan [16,17]. Other suggested interactions are between the chitosan hydrogen bonds, related to NH_2_ groups [18] and C=O groups [19]. In the spectra of the fibers we see interactions involving natural and synthetic polymers: C=O at 1636 cm^−1^ (amide I) and NH at 1534 cm^−1^ (amide II). These bands change their intensity in the hybrid fiber spectra (2, 3, 5 and 6 in Figure 2) in comparison with Ch + S fiber spectra (1 and 4). The bands related to ester groups (1680 and 1230 cm^−1^) and benzene rings (695 cm^−1^) are observed only in the spectra of PET 15 wt% (3 and 6 in Figure 2). One of these bands corresponds to ester group shifts from 1688 cm^−1^ in the spectrum 3 (sample ChS1P2, 15 wt% starch) to 1716 cm^−1^ in the spectrum 6 (sample ChS2P2, 30 wt% starch). This indicates relatively strong interactions of OH groups in starch with ester groups of the PET chains.

### 3.2. Raman Spectroscopy

**Figure 3.**
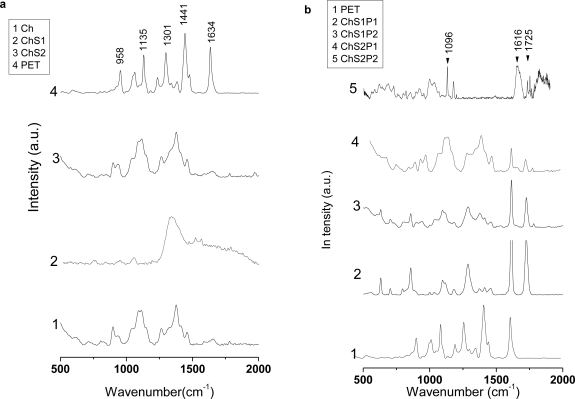
Raman spectroscopy of fibers synthesized by electrospinning. (**a**) Ch, PET, Ch + S and (**b**) hybrids.

Figure 3a shows the Raman spectra of PET, Ch and Ch + S fibers; spectra for ternary fibers are shown in Figure 3b. The spectrum 4 in Figure 3a shows several signals of PET at 1635, 1441, 1301, 1135 and 958 cm^−1^; however, these bands are only related to conformational structures. The typical band for crystalline PET at 1725 cm^−1^ is absent; hence our PET material is amorphous. In the spectra of ternary hybrid fibers (Figure 3b) two bands are detected at 1616 cm^−1^ and 1725 cm^−1^, the first is related to symmetric stretching of carbons 1, 4 of benzene rings and its intensity is a measure of the molecular orientation in the polymeric chains. The second signal is related to the stretching band of C=O groups. This latter signal and the band at 1096 cm^−1^ (stretching C–C associated with ethylene glycol in *trans* conformation) have been correlated with density of crystalline domains [20,21]. These bands are detected also in the hybrid fibers spectra; this shows that originally amorphous PET changes its conformations towards a more ordered structure during the processing. The signals associated with crystal arrangement are clearer in the spectra for samples of lower starch content (15 wt%) (Figure 3b, spectra 2 and 3) than in samples with 30 wt% starch (Figure 3b, spectra 4 and 5).

Ch + S fibers are analyzed in spectra 2 and 3 in Figure 3a. These fibers do not show bands pertaining to crystallinity and preferred molecular orientations. It is important to mention that the crystallinity in hybrid polymers based on chitosan has been associated with several factors. For chitosan solutions prepared with acetic acid, low crystallinity has been reported by Jaworska and colleagues [22]. The same situation has been reported if chitosan is electrospun [23]. Chitosan + poly(vinyl alcohol) hybrids [17] and chitosan + nylon 6 hybrid nanofibers show typical amorphous structures in diffractometry in both systems. Given stretching of molecular chains in the fibers, a sudden solidification at high elongation rates dramatically hinders crystal formation [19].

### 3.3. Morphology from Scanning Electron Microscopy

**Figure 4.**
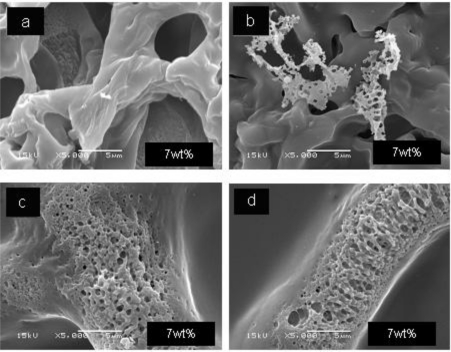
Morphology of chitosan microfibers.

Surface characteristics of electrospun chitosan fibers are presented in Figure 4. These fibers were prepared with 7 wt% chitosan dissolved in aqueous acetic acid solution. Relatively smooth as well as coarse regions are seen. Pores with different diameters are seen. Figures 4b and 4d show scaffold-like structures in the coarse regions. These easily visible differences could be produced by sudden changes in chitosan chain structures during the processing. An interesting explanation was given by Gholipour *et al.* [24]. They observed that increasing the chitosan content produces brittle fibers; also high viscosity of pure chitosan causes severe problems such that fibers cannot even be electrospun. Gholikpour and his colleagues note that an increment in the number of amino groups in acidic media causes a corresponding increase in the density of electrical charges on the surface of the jet, and therefore electrical field effects are stronger [24].

As already noted, the morphology of electrospun ultrafine fibers is influenced by various parameters such as applied voltage, flow rate, distance between capillary and collector, and especially the properties of polymer solution including concentration, surface tension and the nature of the solvent [25]. The effect produced by the content of synthetic polymer and chitosan in the hybrid polymers seems decisive for the fiber morphology.

**Figure 5.**
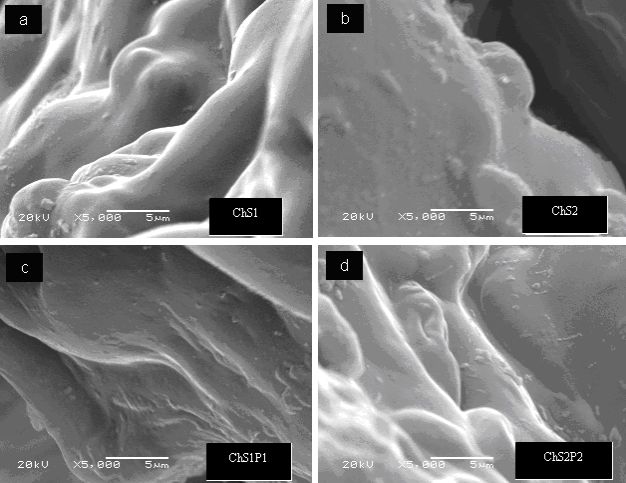
Morphology of ChS fibers: (**a**) ChS1; (**b**) ChS2; hybrid fibers: (**c**) ChS2P1; (**d**) ChS2P2.

Figure 5 shows the SEM images of Ch + S fibers (Figure 5a,b) and Ch + S + PET hybrid fibers (Figure 5c,d). Ch + S fibers prepared with starch 15 wt% (ChS1) are shown in Figure 5a, while fibers containing 30 wt% starch (ChS2) are seen in Figure 5b. Morphology of these fibers is different than that of chitosan fibers (Figure 4). ChS1 and ChS2 have more smooth surfaces, without the presence of brittle or porous regions. This effect is probably caused by the plasticizing behavior of starch. Ternary hybrid fibers do not show large differences from Ch + S fibers, as can be seen in Figure 5c,d. Fibers with 15 wt% PET (ChS2P1) have similar surface characteristics. In the case of fibers with 30 wt% PET (ChS2P2) needle shaped structures are observed, an effect probably produced by the synthetic polymer.

### 3.4. Thermogravimetric Analysis (TGA)

**Figure 6.**
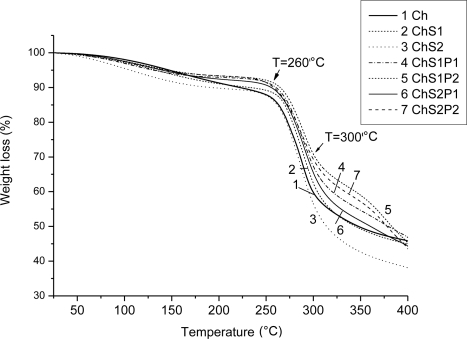
Thermogravimetric analysis (TGA) of Ch, Ch + S fibers and hybrid fibers obtained by electrospinning.

Figure 6 shows thermogravimetric analysis (TGA) diagrams for Ch + S fibers and for ternary hybrid fibers. Two weight loss stages are seen, the first stage is related to decomposition starting at 260 °C and the second stage around 300 °C is associated with oxidative degradation. When starch is incorporated into chitosan to produce Ch + S fibers (thermograms 2 and 3) weight losses observed in both stages are higher than for Ch fibers. However after the incorporation of PET (thermograms 4 to 7), ternary fibers show less weight loss in both stages: decomposition and oxidative degradation. Hybrid fibers which contain more PET (thermograms 5 and 7) show high thermal stability. Thus, the results reveal that the thermal stability in ternary fibers is higher—apparently an effect of addition of the synthetic polymer (PET).

Thermogravimetric analysis of other hybrids based on chitosan has been reported. Those systems also show shifts in the decomposition temperature in comparison with pure chitosan. For instance, in the system chitosan + polycaprolactone the decomposition temperature is between 288–368 °C while for chitosan the respective temperature range is lower, 226–314 °C [16]. Thus, a combination of synthetic and natural polymers provides strong chemical interactions reflected in improved thermal stability.

### 3.5. Differential Scanning Calorimetry

**Figure 7.**
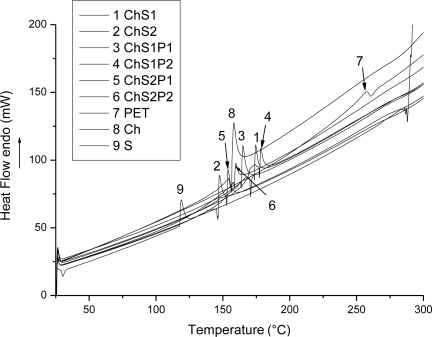
Differential scanning calorimetry (DSC) curves of Ch, S, PET, Ch + S fibers and ternary hybrid fibers synthesized via electrospinning.

Figure 7 shows the DSC diagrams for Ch, S, PET, Ch + S fibers and ternary fibers. We see an endothermic peak attributed to the melting temperature of chitosan fibers T_m_ ≈ 159 °C. T_m_ of starch (powder) and PET (pellets) are located at 119 and 258 °C respectively. Ch + S fibers that contain 15 wt% starch show a T_m_ at 175 °C (curve 1). However, when we have 30 wt% starch (curve 2), the T_m_ decreases to 147 °C, that is towards the melting temperature of pure starch.

DSC results for the ternary hybrids are shown in curves 3 to 6. The analysis of the hybrid materials with 15 wt% starch is shown in the curves 3 and 4. T_m_ ≈ 165 °C for the hybrid with 5 wt% PET (curve 3) and T_m_ ≈ 178 °C for 15 wt% PET (curve 4). Our hybrid fibers with 30 wt% starch show T_m_ ≈ 154 °C and 159 °C for 5 wt% and 15 wt% PET, respectively. Thus T_m_ changes reflect also a success of integration of PET into a system of natural polymers.

DSC is also useful for evaluation of melting enthalpies H_m_ of the analyzed materials. The respective results are 5.33 J/g for electrospun chitosan fibers, 2.66 J/g for the starch (powder) and 7.16 J/g for PET (pellets). The theoretical value of the melting enthalpy for fully crystalline PET is 140 J/g [26]. This also confirms that the PET used in preparation of the hybrid fibers is largely amorphous. Ch + S fibers with 15 wt% starch show H_m_ = 3.51 J/g; this value decreases to 2.7 J/g for 30 wt% starch. We find that the incorporation of starch decreases crystallinity level of Ch + S fibers in comparison with chitosan fibers. When 5 wt% PET is incorporated into the system of 85 wt% chitosan + 15 wt% starch, H_m_ = 4.9 J/g is found. For 15 wt% PET we have H_m_ = 3.8 J/g. The same trend is seen in the hybrid fibers with 30 wt% starch; here the melting enthalpies for 5 wt% and 15 wt% of PET are 4.20 J/g and 3.21 J/g, respectively. Explanation of such results in ternary systems would be difficult. Statistical mechanical theories of polymer-containing ternary systems show complex behavior [27,28]—as do experimental studies of such binary [29] and ternary [30] systems. Some non-equilibrium phases are long living ones, hence a call was made to include such phases in phase diagrams and thus to include in particular glass transitions [29]. For that matter, even binary systems can show complicated s-shaped diagrams of the glass transition temperature T_g_ *vs.* composition [31,32]; explanation of such diagrams is not straightforward.

Table 2 shows the thermal properties of some hybrid systems based on chitosan. In poly(vinyl alcohol) (PVA) + chitosan hybrids, the melting enthalpy increases when the concentration of the synthetic polymer is increased, namely from 27 to 44 J/g [17]. Results for nylon 6 + chitosan hybrids are shown in the same table; nylon has T_m_ ≈ 268 °C. In earlier fibers synthesized by electrospinning, melting temperatures increase with the incorporation of a synthetic polymer [19]. This conclusion applies also to our Ch + S + PET hybrids.

## 4. A Survey of Results

We have demonstrated that the incorporation of PET into chitosan via electrospinning modifies properties in the desired direction. Strong intermolecular interactions and compatibility between the synthetic and natural polymers created during the synthesis via electrospinning are verified by IR analysis. The orientation achieved is the result of the interactions between natural and synthetic polymers and the electrospinning process. Molecular interactions are also reflected in morphology and thermal properties of the hybrids.

Development of hybrid materials such as ours can have a number of applications. The hybrids based on chitosan and PET might provide stronger biodegradability and biocompatibility—in addition to satisfactory mechanical and thermal characteristics. Synthetic + natural hybrids based on chitosan via electrospinning allow creation of new materials with advantageous features from both kinds of polymers—an alternative to the conventional polymers.

## Figures and Tables

**Table 1.** Concentrations and nomenclature of Ch + S + PET hybrid fibers synthesized using electrospinning.

**Blends (wt%)**			**Hybrid Fibers Nomenclature**
**Blend 1**		**Blend 2**	

**Chitosan**	**Starch**	**PET**	
		0	ChS1
85	15	5	ChS1P1
		15	ChS1P2

		0	ChS2
70	30	5	ChS2P1
		15	ChS2P2

**Table 2.** Thermal properties in different hybrid systems based on chitosan synthesized by electrospinning.

**Hybrid Polymer**	**Weight Ratio (wt/wt)**	**Melting Point T_m_ (°C)**	**Melting Enthalpy (J/g)**	**Reference**
PVA + Ch	100/0	199	54.3	[17]
	90/10	194	44.0	
	80/20	192	42.8	
	75/25	190	27.0	

Nylon 6 + Ch	100/0	268	-	[19]
	90/10	265	-	
	85/15	261	-	
	80/20	260	-	
	75/25	258	-	

Ch+ S + PET	85-15/0	175	3.51	
	70-30/0	147	2.70	
	85-15/5	165	4.90	
	85-15/15	178	3.80	
	70-30/5	154	4.20	
	70-30/15	159	3.21	
